# Radiative cooling of H_3_O^+^ and its deuterated isotopologues†
†Electronic supplementary information (ESI) available. See DOI: 10.1039/c6cp04661d



**DOI:** 10.1039/c6cp04661d

**Published:** 2016-09-01

**Authors:** Vladlen V. Melnikov, Sergei N. Yurchenko, Jonathan Tennyson, Per Jensen

**Affiliations:** a Siberian Institute of Physics & Technology , Tomsk State University , Tomsk , 634050 , Russia; b Department of Physics & Astronomy , University College London , London WC1E 6BT , UK . Email: s.yurchenko@ucl.ac.uk; c Physikalische und Theoretische Chemie , Fakultät für Mathematik und Naturwissenschaften , Bergische Universität Wuppertal , 42097 Wuppertal , Germany

## Abstract

In conjunction with *ab initio* potential energy and dipole moment surfaces for the electronic ground state, we have made a theoretical study of the radiative lifetimes for the hydronium ion H_3_O^+^ and its deuterated isotopologues. We compute the ro-vibrational energy levels and their associated wavefunctions together with Einstein coefficients for electric dipole transitions. A detailed analysis of the stability of the ro-vibrational states has been carried out and the longest-living states of the hydronium ions have been identified. We report estimated radiative lifetimes and cooling functions for temperatures <200 K. A number of long-living meta-stable states are identified, capable of population trapping.

## Abstract 2

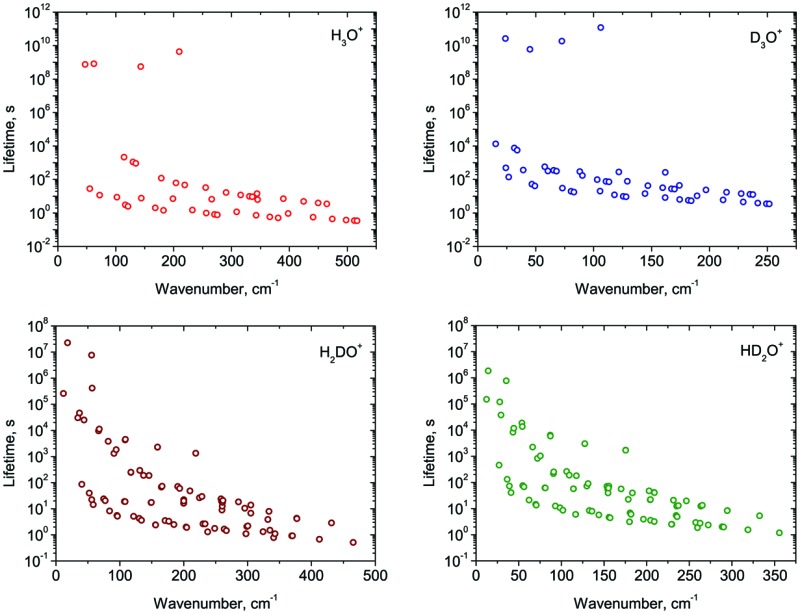
In conjunction with *ab initio* potential energy and dipole moment surfaces for the electronic ground state, we have made a theoretical study of the radiative lifetimes for the hydronium ion H_3_O^+^ and its deuterated isotopologues.

## 1 Introduction

In the Universe, molecules are found in a wide variety of environments: from diffuse interstellar clouds at very low temperatures to the atmospheres of planets, brown dwarfs and cool stars which are significantly hotter. In order to describe the evolution of the diverse, complex environments, it is essential to have realistic predictions of the radiative and cooling properties of the constituent molecules. Such predictions, in turn, require reasonable models for the energetics of each molecular species present.

Although interstellar molecular clouds are usually characterised as cold, they are mostly not fully thermalized. Whether a species attains thermal equilibrium with the environment depends on the radiative lifetimes of its states and the rate of collisional excitations to the states: this is normally characterised by the critical density. In non-thermalized regions, radiative lifetimes are also important for modelling the maser activity observed for many species. The long lifetimes associated with certain excited states can lead to population trapping and non-thermal, inverted distributions. Such unexpected state distributions have been observed for the H_3_^+^ molecule both in space^1,2^ and in the laboratory.^3,4^

Dissociative recombination of hydronium H_3_O^+^ has been extensively studied in ion storage rings.^5–9^ The lifetimes calculated in the present work suggest that H_3_O^+^ and its isotopologues will exhibit population trapping in a manner similar to that observed for H_3_^+^ in storage rings. Dissociative recombination of hydronium has been postulated as a possible cause of emissions from super-excited water in cometary comae^10^ and as the mechanism for a spontaneous infrared water laser.^11^

Hydronium and its isotopologues play an important role in planetary and interstellar chemistry.^7,12^ These molecular ions are found to exist abundantly in both diffuse and dense molecular clouds as well as in comae. Moreover, H_3_O^+^ is a water indicator and can be used to estimate water abundances when direct detection is unfeasible.^13^ Consequently, the ions have been the subject of numerous theoretical and experimental studies (see, for example, ref. 7, 8, 12–46 and references therein) mainly devoted to the spectroscopy and chemistry of the species.

Whereas the cooling function of the H_3_^+^ ion has been extensively studied by Miller *et al.*,^47–49^ no information about the radiative and cooling properties of H_3_O^+^ and its deuterated isotopologues has been available thus far. In the present work, we remedy this situation by determining theoretically the ro-vibrational states of the ions H_3_O^+^, H_2_DO^+^, HD_2_O^+^, and D_3_O^+^. We use *ab initio* potential energy (PES) and dipole moment surfaces (DMS) for the ground electronic states of H_3_O^+^ from ref. 50 to compute for each of the four ions considered here, ro-vibrational energy levels, the accompanying wavefunctions, and Einstein coefficients for the relevant ro-vibrational (electric dipole) transitions by means of the nuclear-motion program TROVE.^51^ Lifetimes of individual ro-vibrational states are calculated and analyzed together with the overall cooling rates. Recently, the same methodology was used to estimate the sensitivities of hydronium-ion transition frequencies to a possible time variation of the proton-to-electron mass ratio.^50^

We present a detailed analysis of the stability of the ro-vibrational states of the hydronium ions and identify the states with the longest lifetimes. This study is based on the methodology^52^ developed very recently as part of the ExoMol project.^53^ The ExoMol project aims at a comprehensive description of the spectroscopic properties of molecules important for atmospheres of exoplanets and cool stars. The molecular lifetimes and cooling functions determined for H_3_O^+^ and its deuterated isotopologues in the present work are available in the new ExoMol data format.^54^

## 2 Theory and computation

The ro-vibrational energy levels and wavefunctions of the ions under study were calculated variationally using the TROVE program^51,55^ in a manner similar to the successful calculations previously carried out for several other XY_3_ pyramidal molecules^56–61^ including ammonia NH_3_ (see ref. 62 and 63), which exhibits the same large-amplitude, ‘umbrella-flipping’ inversion motion as H_3_O^+^. The inversion barrier of H_3_O^+^ is 650.9 cm^–1^,^64^ which is lower than 1791 cm^–1^ found for NH_3_.^39^ As a result, H_3_O^+^ inversion splitting of the ro-vibrational ground state, 55.35 cm^–1^ (ref. 65), is significantly larger than 0.793 cm^–1^ (ref. 39) of NH_3_.

We have used *ab initio* PES and DMS of H_3_O^+^ (ref. 50), computed at the MRCI/aug-cc-pwCV5Z(5Z) and MRCI/aug-cc-pwCVQZ(QZ) levels of theory, respectively. Complete basis set (CBS) extrapolation was used to obtain the Born–Oppenheimer PES (see ref. 50 for details).

The basis set used in the variational computations of the ro-vibrational states is defined in ref. 51. In all calculations of the present work, the orders of kinetic and potential energy expansions were set to 6 and 8, respectively. We used Morse-type basis functions for the stretching modes and numerical basis functions (numerical solutions of the corresponding 1D problem obtained within the framework of the Numerov–Cooley scheme^66^) for the bending vibrations. The *ab initio* equilibrium structure of H_3_O^+^ is characterized by an O–H bond length of 0.9758 Å and a H–O–H angle of 111.95°. The vibrational basis set is controlled by the polyad number defined by

1 *P* = 2(*v*_1_ + *v*_2_ + *v*_3_) + *v*_4_ + *v*_5_ + *v*_6_/2,

where *v*_1_, *v*_2_, *v*_3_ represent the quanta of the stretching motion, *v*_4_ and *v*_5_ are those of the asymmetric bending motion and *v*_6_ is that of the primitive basis set functions for the inversion. In the present work, the maximum polyad number *P*_max_ (where *P* < *P*_max_) was set to 28. The ro-vibrational basis sets used for H_3_O^+^ and D_3_O^+^ were symmetrized according to the 𝒟_3h_(*M*) molecular symmetry group,^67^ while the calculations for the asymmetric isotopologues H_2_DO^+^ and HD_2_O^+^ were done under the 𝒞_2v_(*M*) symmetry. The computational details of the basis set construction can be found in ref. 62 as well as the details of the calculations of the Einstein coefficients *A*_*if*_. The latter were computed for all possible initial, *i*, and final, *f*, states lying less than 600 cm^–1^ above the zero point energy with *J* ≤ 7. According to our estimations, this should be sufficient to describe the populations of the ro-vibrational states at thermodynamic temperatures up to 200 K. In this work we concentrate on the low energy applications. Higher temperatures would require higher rotational excitations *J* and therefore more involved calculations.

The lifetimes of the states were computed as^52^
2
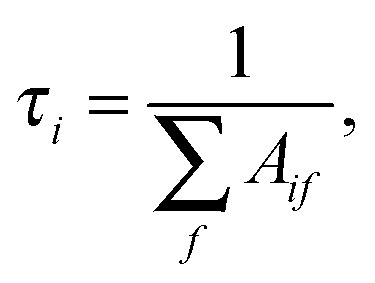
where the summation is taken over all possible final states *f* with energies lower than that of the given initial state *i*. The Einstein coefficients (in units of s^–1^) are defined as follows:^68^
3
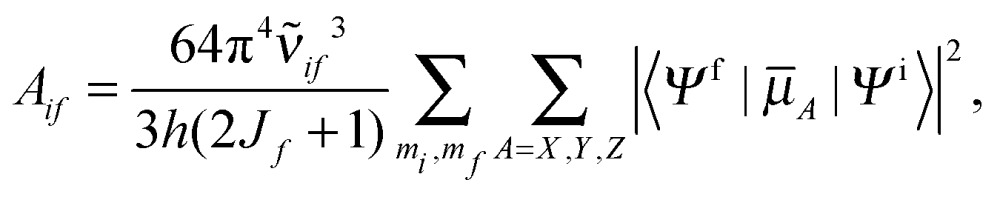
where *h* is Planck's constant, *ν̃*_*if*_ (cm^–1^) is the wavenumber of the line, (*hcν̃*_*if*_ = *E*_*f*_ – *E*_*i*_), *J*_*f*_ is the rotational quantum number for the final state, *Ψ* ^*i*^ and *Ψ* ^*f*^ represent the ro-vibrational eigenfunctions of the final and initial states, respectively, *m*_*i*_ and *m*_*f*_ are the corresponding projections of the total angular momentum on the *Z* axis, and *μ̄*_*A*_ is the electronically averaged component of the dipole moment (Debye) along the space-fixed axis *A* = *X*, *Y*, *Z* (see also ref. 69).

The cooling function *W*(*T*) is the total power per unit solid angle emitted by a molecule at temperature *T*; it is given by the following expression:^52^
4
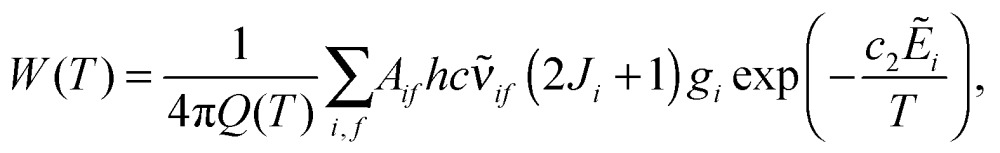
where *g*_*i*_ is the nuclear spin statistical weight factor of the state *i*. In the Boltzmann factor exp(–*c*_2_ **_*i*_/*T*), **_*i*_ (=*E*_*i*_/*hc*) is the term value of state *i* and *c*_2_ = *hc*/*k* is the second radiation constant (*k* is the Boltzmann constant). The partition function *Q*(*T*) is defined as5
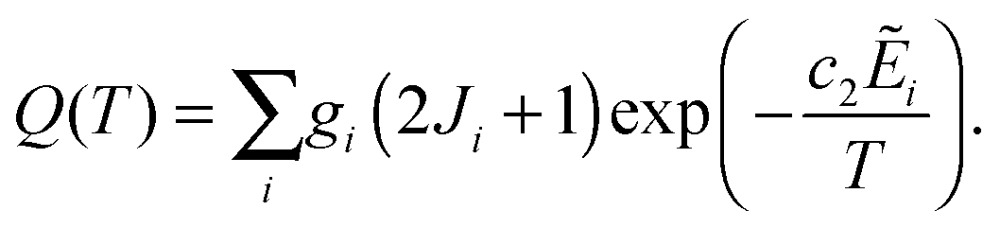
 Partition functions were computed for each ion employing the sets of ro-vibrational energies obtained with the chosen basis set.

The same PES and DMS were used for each isotopologue, which means that no allowance was made for the breakdown of the Born–Oppenheimer approximation. The energies of H_3_O^+^ and the three deuterated isotopologues are very different not only due to the mass changes, but also due to the different symmetries these species belong to and the ensuing nuclear spin statistics. We use the molecular symmetry group^67^ 𝒟_3h_(*M*) to classify the ro-vibrational states of the highest-symmetry species H_3_O^+^ and D_3_O^+^, and 𝒞_2v_(*M*) for the lower-symmetry isotopologues H_2_DO^+^ and HD_2_O^+^. The differences in the Einstein coefficients are also quite substantial, especially between the 𝒟_3h_(*M*) and the 𝒞_2v_(*M*) isotopologues. In general, isotope substitution in ions often leads to large changes in ro-vibrational intensities. This is because these intensities depend on the components of the electric dipole moment in the molecule-fixed axis system which, by definition, has its origin in the nuclear center-of-mass. Upon isotopic substitution, the center-of-mass, and thus the origin of the molecule-fixed axis system, are displaced. For a neutral molecule (*i.e.*, a molecule with no net charge) this does not change the dipole moment components but, for an ion, they do (see, for example, ref. 70). Owing to intensities and Einstein coefficients changing much upon isotopic substitution, the lifetimes of the ro-vibrational states are expected to vary strongly with isotopologue for H_3_O^+^. Selection rules for *J* are

6 *J*′ – *J*′′ = 0, ±1 and *J*′ + *J*′′ > 0.

The symmetry selection rules for H_3_O^+^ and D_3_O^+^ are

7 *A*_1_′ ↔ *A*_1_′′, *A*_2_′ ↔ *A*_2_′′, *E*′ ↔ *E*′′

where, for H_3_O^+^ levels of *A*_1_′ and *A*_1_′′ symmetry are missing^67^ so that the associated selection rule is irrelevant, while those for H_2_DO^+^ and HD_2_O^+^ are

8 *A*_1_ ↔ *A*_2_, *B*_1_ ↔ *B*_2_.

Nuclear spin statistics^67^ result in three distinct spin species of D_3_O^+^, *ortho* (ro-vibrational symmetry *A*_1_′ or *A*_1_′′, nuclear spin statistical weight factor *g*_ns_ = 10), *meta* (*E*′ or *E*′′, *g*_ns_ = 8) and *para* (*A*_2_′ or *A*_2_′′, *g*_ns_ = 1). As mentioned above, *A*_1_′ and *A*_1_′′ ro-vibrational states are missing for H_3_O^+^ and there are only *ortho* (*A*_2_′ or *A*_2_′′, *g*_ns_ = 4) and *para* (*E*′ or *E*′′, *g*_ns_ = 2) states. The 𝒞_2v_(*M*) isotopologues H_2_DO^+^ and HD_2_O^+^ have *ortho* and *para* states: for H_2_DO^+^, *B*_1_ and *B*_2_ states are *ortho* with *g*_ns_ = 9, and *A*_1_ and *A*_2_ states are *para* with *g*_ns_ = 3. For HD_2_O^+^, the *ortho*–*para* states are interchanged relative to H_2_DO^+^: *A*_1_ and *A*_2_ states are now *ortho* with *g*_ns_ = 12 whereas *B*_1_ and *B*_2_ states are *para* with *g*_ns_ = 6.

## 3 Results and discussion

In order to validate our description of the energetics of H_3_O^+^ and its deuterated isotopologues, we compare in Table 1 calculated vibrational energies for these molecules with the available, experimentally derived values. In view of the fact that the calculations are based on a purely *ab initio* PES, the agreement between theory and experiment is excellent. The results suggest that the *ab initio* data used in the present work, in conjunction with the variational TROVE solution of the ro-vibrational Schrödinger equation, are more than adequate for obtaining accurate lifetimes of the molecules studied.

**Table 1:** Available, experimentally derived vibrational term values of H_3_O^+^ and its deuterated isotopologues (in cm^–1^) compared to theoretical values from the present work

State	Sym.	Exp.	Ref.	Calc.	Exp.–Calc.
H_3_O^+^					
*ν*_2_^+^	*A*_1_	581.17	26	579.07	2.10
2*ν*_2_^+^	*A*_1_	1475.84	25	1470.67	5.17
*ν*_1_^+^	*A*_1_	3445.01	65	3442.61	2.40
*ν*_3_^+^	*E*	3536.04	65	3532.58	3.46
*ν*_4_^+^	*E*	1625.95	27	1623.02	2.93
0^–^	*A*_1_	55.35	65	55.03	0.32
*ν*_2_^–^	*A*_1_	954.40	26	950.94	3.46
*ν*_1_^–^	*A*_1_	3491.17	65	3488.32	2.85
*ν*_3_^–^	*E*	3574.29	65	3571.04	3.25
*ν*_4_^–^	*E*	1693.87	27	1690.65	3.22

D_3_O^+^					
*ν*_2_^+^	*A*_1_	453.74	31	451.58	2.16
*ν*_3_^+^	*E*	2629.65	31	2627.14	2.51
0^–^	*A*_1_	15.36	71	15.38	–0.02
*ν*_2_^–^	*A*_1_	645.13	31	642.79	2.34
*ν*_3_^–^	*E*	2639.59	31	2637.10	2.49

H_2_DO^+^					
0^–^	*B*_1_	40.56	43	40.39	0.17
*ν*_1_^+^	*A*_1_	3475.97	42	3473.27	2.70
*ν*_1_^–^	*B*_1_	3508.63	42	3505.51	3.12
*ν*_3_^+^	*B*_2_	3531.50	42	3528.07	3.43
*ν*_3_^–^	*A*_2_	3556.94	42	3553.63	3.31

HD_2_O^+^					
0^–^	*B*_1_	26.98	43	26.92	0.06

**Fig. 1:**
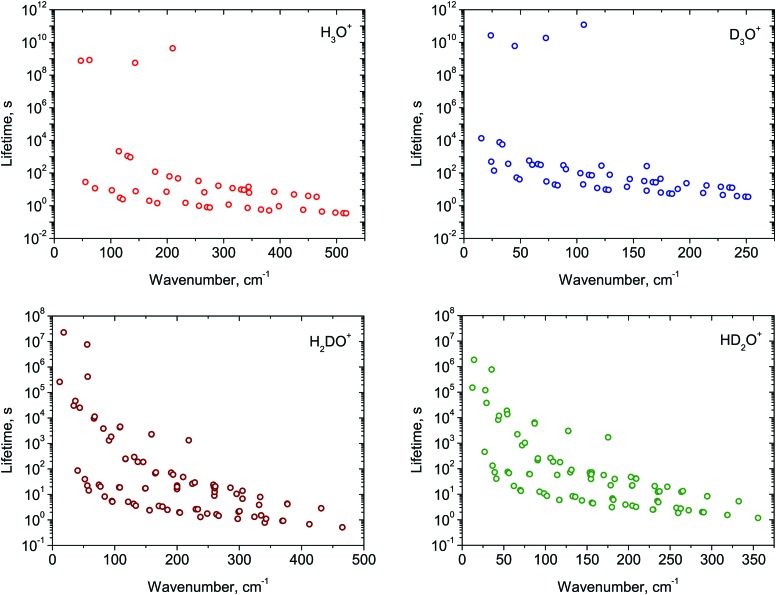
Calculated lifetimes *τ* of the ro-vibrational states (*J* ≤ 7) of H_3_O^+^ and its deuterated isotopologues. The lifetime values are plotted logarithmically.

In Fig. 1, we plot the lifetimes calculated for the ro-vibrational states of H_3_O^+^ and its deuterated isotopologues against the associated term values ** (*J* ≤ 7, ** < 600 cm^–1^). In general, the lifetimes exhibit the expected gradual decrease with increasing term value. The complete list of lifetimes for all four isotopologues is given as ESI† to this paper.

Lifetimes *τ* of the longest-lived states of the ions are compiled in Table 2. The lowest-lying state of each of the nuclear spin species *ortho* and *para* (and *meta* for D_3_O^+^) has an infinitely long lifetime; it has no state to decay to.

**Table 2:** Lifetimes *τ* for the longest-lived states of the H_3_O^+^, D_3_O^+^, H_2_DO^+^, and HD_2_O^+^ ions. All states listed are rotational states in the vibrational ground state (*i.e.*, the inversion state 0^+^). The states are labeled (*J*,*K*,*Γ*) and (*J*_*K*_a_,*K*_c__,*Γ*) for 𝒟_3h_(*M*) and 𝒞_2v_(*M*) isotopologues, respectively, with *Γ* as the symmetry of the state in the respective group

State	Term value, cm^–1^	*τ*
H_3_O^+^		(Years)
(1,0,*A*_2_′)	22.47	∞
(1,1,*E*′′)	17.38	∞
(3,3,*A*_2_′′)	88.96	∞
(5,5,*E*′′)	209.58	140.1
(2,1,*E*′′)	62.29	26.2
(2,2,*E*′)	47.03	23.9
(4,4,*E*′)	143.15	17.7

D_3_O^+^		(Years)
(0,0,*A*_1_′)	0.00	∞
(1,0,*A*_2_′)	11.33	∞
(1,1,*E*′′)	8.78	∞
(3,3,*A*_2_′′)	45.02	∞
(5,5,*E*′′)	106.15	3816.0
(2,2,*E*′)	23.79	857.1
(4,4,*E*′)	72.48	594.4
(3,3,*A*_1_′′)	45.02	190.4

H_2_DO^+^		(Days)
(0_0,0_,*A*_1_)	0.00	∞
(1_1,1_,*B*_1_)	15.70	∞
(1_1,0_,*B*_2_)	18.07	265.4
(2_2,1_,*A*_2_)	55.82	89.1
(2_2,0_,*A*_1_)	56.60	4.8
(1_0,1_,*A*_2_)	11.69	3

HD_2_O^+^		(Days)
(0_0,0_,*A*_1_)	0.00	∞
(1_1,1_,*B*_1_)	9.53	∞
(1_1,0_,*B*_2_)	14.24	21.6
(2_2,1_,*A*_2_)	35.35	8.9
(1_0,1_,*A*_2_)	12.19	1.8
(2_2,0_,*A*_1_)	27.77	1.4

Low-lying, purely rotational states with low *J* values have the longest lifetimes; they have the smallest numbers of decay channels and/or the lowest probability for spontaneous emission transitions. The higher-symmetry species H_3_O^+^ and D_3_O^+^ (with 𝒟_3h_(*M*) symmetry) have more restrictive selection rules than the 𝒞_2v_(*M*) species HD_2_O^+^ and H_2_DO^+^, and so H_3_O^+^ and D_3_O^+^ states live significantly longer (typically tens to hundreds of years) compared to the day-long lifetimes of HD_2_O^+^ and H_2_DO^+^. Thus, D_3_O^+^ has three meta-stable states with lifetimes longer than 100 years. The longest-lived of these, with *τ* = 3816 years, is the rotational state (*J* = 5, *K* = 5, *E*′′) of the vibrational ground state. In comparison, the longest-lived meta-stable state of H_2_DO^+^ the rotational state (*J*_*K*_a_,*K*_c__,*Γ*) = (1_1,0_,*B*_2_) has a lifetime of 265 days.

**Fig. 2:**
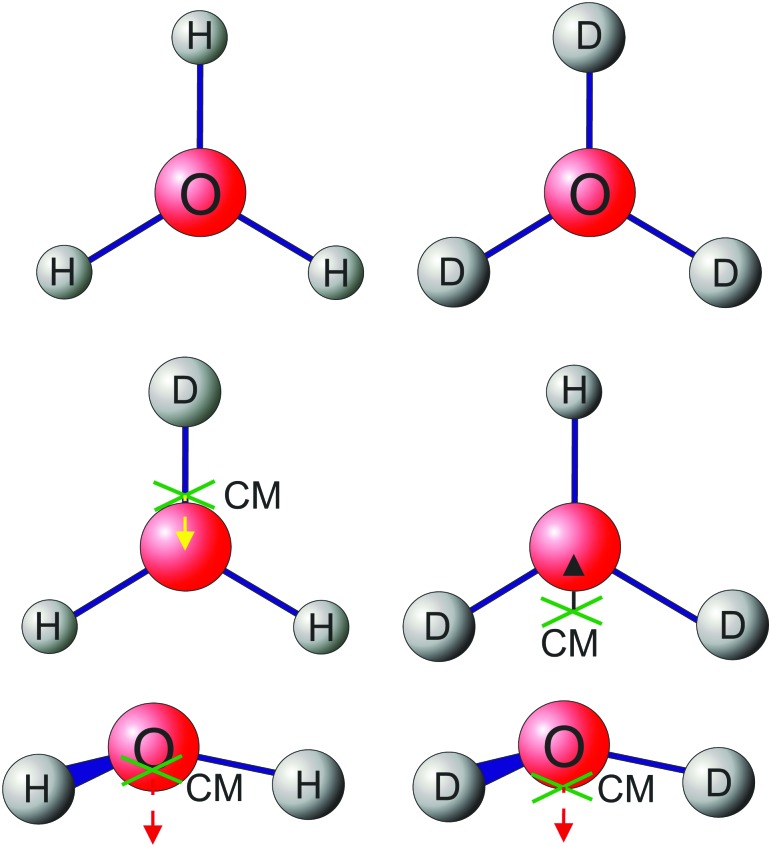
Displacements of the center-of-mass (green crosses) upon deuteration of H_3_O^+^. Arrows indicate the dipole moment components.

As mentioned above, the symmetry lowering from 𝒟_3h_(*M*) to 𝒞_2v_(*M*) gives rise to another important effect illustrated in Fig. 2. For H_3_O^+^ and D_3_O^+^ both the nuclear center-of-mass and the nuclear center-of-charge lie on the *C*_3_ symmetry axis for nuclear geometries with 𝒞_3v_ geometrical symmetry.^67^ We take the *C*_3_ axis to be the *z* axis of the molecule-fixed axis system whose origin, by definition, is at the nuclear center-of-mass. Consequently, at 𝒞_3v_-symmetry geometries the dipole moment lies along the *z* axis and its *x*- and *y*-components vanish. The non-zero *z*-component is responsible for the parallel bands in the spectra of these species, including the rotation-inversion band^44^ (the pure rotation band is forbidden by symmetry). Botschwina *et al.* ^18^ estimated the corresponding transition dipole for the inversion 0^–^ ↔ 0^+^ band to be 1.47 D. Our *ab initio* value is slightly higher, 1.80 D. For H_2_DO^+^ and HD_2_O^+^, the center-of-charge obviously is unchanged relative to H_3_O^+^ and D_3_O^+^ but the center-of-mass is shifted, and this produces a non-vanishing perpendicular dipole moment at 𝒞_3v_-symmetry geometries. If we take the *x* axis to be in the plane defined by the *C*_3_ symmetry axis^67^ and the O–H bond for HD_2_O^+^, and in the plane defined by the *C*_3_ symmetry axis and the O–D bond for H_2_DO^+^, then HD_2_O^+^ and H_2_DO^+^ acquire non-vanishing *x* dipole moment components. Therefore the perpendicular transitions (Δ*K*_c_ = ±1) of H_2_DO^+^ and HD_2_O^+^ in the vibrational ground state are much stronger than the Δ*K* = ±1 transitions of H_3_O^+^ and D_3_O^+^ which are caused by intensity stealing from the vibrational spectrum.^67^ Besides, this *x* component is larger for HD_2_O^+^ than for H_2_DO^+^ owing to the greater displacement of the nuclear center-of-mass. This is probably why the HD_2_O^+^ lifetimes are shorter on the average than those of H_2_DO^+^. The *z* dipole moment component also changes with isotopic substitutions, see Fig. 2.

The longest-living states of D_3_O^+^ (Table 2) have lifetimes about 27 times longer than those of H_3_O^+^. Presumably, this is mainly caused by the fact that D_3_O^+^ has lower ro-vibrational term values than H_3_O^+^. Thus, D_3_O^+^ has lower values of *ν̃*_*if*_ in eqn (3) and this, in turn, causes lower Einstein-*A* coefficients and higher values of *τ* [eqn (2) and (3)].

**Fig. 3:**
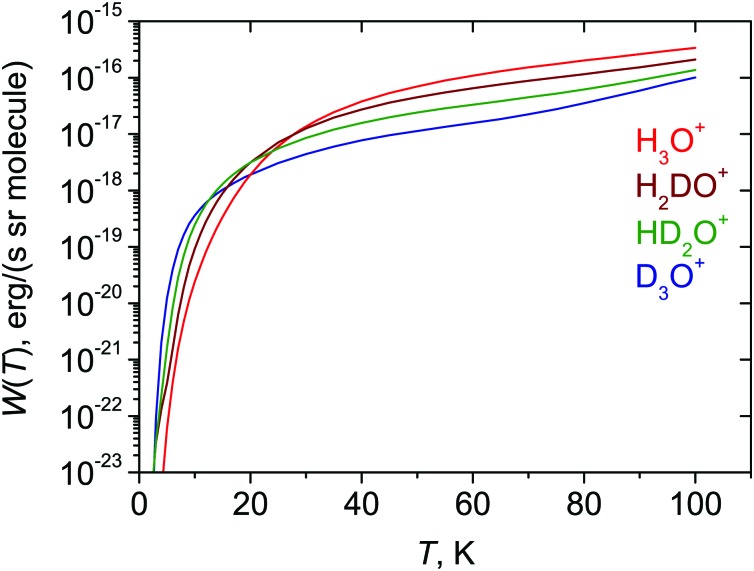
Calculated cooling functions of H_3_O^+^ and isotopologues.

The calculated radiative cooling functions *W*(*T*) [eqn (4)] for H_3_O^+^ and its deuterated isotopologues are shown in Fig. 3. At temperatures above 30 K the cooling decreases with increasing numbers of deuterium atoms in the molecule. This can be easily understood from eqn (3) and (4): *W*(*T*) is proportional to *ν̃*_*if*_^4^, and *ν̃*_*if*_ is approximately inversely proportional to the mass of hydrogen for the rotational states populated at the temperatures considered. Therefore, at moderate and high temperatures the lighter isotopologues are better coolers. However, Fig. 3 shows that at lower temperatures their roles change and the deuterated species become the better coolers. This is because the term values of the lowest, infinite-lifetime (and therefore coldest) states vary as 47.03 cm^–1^ (2,2,*E*′,0^+^), 23.79 cm^–1^ (2,2,*E*′,0^+^), 12.19 cm^–1^ (1_0,1_^+^,*A*_2_), and 11.69 cm^–1^ (1_0,1_^+^,*A*_2_) for H_3_O^+^, D_3_O^+^, HD_2_O^+^, and H_2_DO^+^, respectively. At very low temperatures, the molecules will tend to collect in the lowest accessible state, and the higher the term value of this state, the more difficult it is to cool the isotopologue in question. Because of this, for example, it is more difficult to cool H_3_O^+^ than D_3_O^+^ at temperatures below 30 K.

## 4 Conclusions

We have carried out a theoretical study of the ro-vibrational states of the hydronium ion H_3_O^+^ and its deuterated isotopologues. *Ab initio* potential energy and electric dipole moment surfaces were used to calculate ro-vibrational energy levels, corresponding wavefunctions and Einstein coefficients for the low-lying ro-vibrational transitions of these ions. We have analyzed the stability of the ro-vibrational states and computed the radiative lifetimes and cooling functions for temperatures below 200 K.

Taking into account only spontaneous emission as a cause of decay of ro-vibrational states (and neglecting collisions and stimulated emission) we find the longest-lived hydronium state for D_3_O^+^: the population in the rotational state with (*J*,*K*,*Γ*) = (5,5,*E*′′) is trapped for 3816.0 years, which is relatively ‘hot’ (152 K), at least in the context of molecular cooling, for example in storage rings. In this work we have identified a number of relatively hot (*E*/*k* > 100 K) meta-stable states with a lifetime longer than 10 s (typical timescales of ion storage experiments). Such meta-stable states which will be populated and hamper the cooling of hydronium ions to a temperature of a few Kelvin. The molecule with the shortest-lived meta-stable states is HD_2_O^+^ with lifetimes of a few days. The timescale of interstellar collisions in diffuse clouds is longer (about a month), and thus some of these states undergo spontaneous emission.

Our calculations show that deuteration influences significantly the hydronium lifetimes. This effect is mostly caused by the symmetry lowering from 𝒟_3h_(*M*) to 𝒞_2v_(*M*) and the ensuing perpendicular dipole moment component. A number of long-living meta-stable states are identified, capable of population trapping. Compared to the deuterated species, the cooling of the lightest isotopologue H_3_O^+^ is most efficient at higher temperatures (*T* > 30 K). However, this changes at very low temperatures where the H_3_O^+^ ions are trapped at relatively high energy.

The results obtained can be used to assess the cooling properties of the hydronium ion in ion storage rings and elsewhere.
